# The Survival Response of Earthworm (*Eisenia fetida* L.) to Individual and Binary Mixtures of Herbicides

**DOI:** 10.3390/toxics10060320

**Published:** 2022-06-12

**Authors:** Elham Samadi Kalkhoran, Mohammad Taghi Alebrahim, Hamid Reza Mohammaddoust Chamn Abad, Jens Carl Streibig, Akbar Ghavidel, Te-Ming Paul Tseng

**Affiliations:** 1 Department of Plant Production and Genetics, Faculty of Agriculture and Natural Resources, University of Mohaghegh Ardabili, Ardabil 56199-11367, Iran; samadielham@uma.ac.ir (E.S.K.); hr_chamanabad@uma.ac.ir (H.R.M.C.A.); 2 Department of Plant and Environmental Science, Faculty of Life Sciences, University of Copenhagen, DK-2360 Copenhagen, Denmark; jcs@plen.ku.dk; 3 Department of Soil Science and Engineering, Faculty of Agriculture and Natural Resources, University of Mohaghegh Ardabili, Ardabil 56199-11367, Iran; ghavidel@uma.ac.ir; 4 Department of Plant and Soil Sciences, Mississippi State University, Starkville, MS 39762, USA; t.tseng@msstate.edu

**Keywords:** ecotoxicology, isobole method, risk assessment, soil invertebrates

## Abstract

Frequent use of herbicides may impose a risk on non-target species. The objective was to test the combined toxic effect of binary herbicide mixtures—metribuzin:halosulfuron and metribuzin:flumioxazin—on non-target earthworms in two test systems: filter paper and a soil toxicity test system. The joint action experiments were independently run twice to substantiate the findings. The most potent individual herbicide was metribuzin, with a 50% lethal concentration (LC_50_) of 17.17 µg ai. cm^−2^ at 48 h in the filter paper test. The toxicity of the individual herbicides on the filter paper test was ranked as metribuzin>halosulfuron>flumioxazin. In the soil test, metribuzin and halosulfuron had high toxicity with an LC_50_ of 8.48 and 10.08 mg ai. kg^−1^, respectively, on day 14. Thus, the individual herbicide ranking did not change between the filter paper and artificial soil tests. The herbicide’s mixed effect in both test systems showed a consistent antagonistic effect relative to a Concentration Addition reference model. It indicates that the mixtures retracted the herbicide’s action in the earthworms.

## 1. Introduction

Weed infestation reduces yields and product quality and increases production costs [1]. One of the most efficient tools to control weeds is the use of selective herbicides [2,3]. The purpose of using herbicides is to prevent competition between weeds and crops [4]. According to [5], herbicides represent the highest load of active ingredients per ha than any other pesticides. Using herbicides either alone or in mixtures might effectively control weeds but may affect non-target organisms within and outside the treated field. Less than 0.1% of a herbicide often reaches the target plants, and the remainder is absorbed by the crop, left in the soil, or contaminates the environment [6]. Soil-dwelling animals such as earthworms might be affected, even though the herbicide’s site of action is not targeted towards animals [7]. Earthworms are important macroinvertebrates [8] that make up more than 80% of the terrestrial invertebrate’s biomass. They have critical functions in soil structure, soil characteristics (pH, organic matter, nitrogen, and granulometry), nutrient immobilization, nitrogen mineralization of organic matter, soil permeability, and microbial community activity [9]. They are used as test species to measure the biological effect of heavy metals and pesticide residues in soil due to their high sensitivity to soil pollutions [10,11].

The *Eisenia fetida* is currently used as test species in ecotoxicology [12], probably because it is easily raised and bred in the laboratory and, therefore, a common species in laboratory experiments. Finally, the use of it is recommended in ecotoxicological studies by European Union (EU), Organisation for Economic Co-operation and Development (OECD), and U.S. Environmental Protection Agency (EPA) [13,14,15].

Herbicide mixtures are commonly used to control diverse weed floras in crops because mixtures widen the control spectrum [16,17]. The pesticide mixtures have received much attention in recent decades, particularly their effect outside the arable land [18,19]. The joint action of herbicide mixtures is generally being analyzed by either Concentration Addition (CA) or Independent Action (IA) reference models [20]. The two models cover various views on the intrinsic mechanism of joint action of compounds in an organism.

This paper solely focuses on CA, which assumes that two compounds do not interfere with each other in an organism [21]. Biochemically, they do not interfere at the target enzyme binding site or impair or enhance each other’s uptake and translocation in the plant. CA requires knowing the relative potency (strength) at any response level between the two herbicides in a mixture, applied singly. If the effect of binary mixtures at any response level, say LC_50_ (lethal concentration that kills 50% of the test animals), in fixed ratios diverts from the CA LC_50_ Isobole, the effect is classified as either antagonistic or synergistic, respectively [2,16,22]. If a mixture follows the CA isobole, viz. follows straight-line CA isoboles, the herbicides do not affect each other’s action [23] in *Eisenia fetida.* If the mixtures do not follow CA, they may be acting synergistically or antagonistically.

It is important to note that most herbicides do not affect animals as their primary modes of action solely target plant metabolism, not existing in animals. Those herbicides can either be applied in mixtures to control important weed species or be sequentially applied. In either case, the joint action of mixtures is of interest to agronomists and ecotoxicology in unraveling the joint action on non-target species.

The objective of this paper was to evaluate the acute LC_50_ of the herbicides either alone or in binary mixtures of metribuzin:halosulfuron and metribuzin:flumioxazin by isobole method.

## 2. Materials and Methods

### 2.1. Test Organism

The Iran earthworm company delivered the *Eisenia fetida*. They were carefully transferred to the laboratory in moist soil. Under laboratory conditions, the earthworms were kept for five days in boxes containing the original medium (a mixture of sand, clay loam soil, and peat (1:1:1 *v*/*v*)) and cattle manure free of veterinarian products, and maintained at room temperature (20 ± 1 °C). The moisture content of the medium was 35%. The soil water content was measured every week, and moisture was adjusted to 35% of the maximum water-holding capacity by adding distilled water. Healthy and clitellate adult earthworms (weight of 350–600 mg) with completely developed clitella were used in toxicity tests [24].

### 2.2. Herbicides

Formulated metribuzin (Sencor, Wettable Powder (WP) 70%) was obtained from Bayer, Persian AG, Tehran, Iran, halosulfuron (Sempra, Water dispersible Granules (WG) 75%) from Nufarm Company, Iranian Research Institute of Plant Protection, Tehran, Iran and flumioxazin (Pledge, Wettable Powder (WP) 50%) was from Sumitomo Chemical Company, Iranian Research Institute of Plant Protection, Tehran, Iran. Metribuzin, halosulfuron, and flumioxazin are used in potatoes [16,25,26,27]. Metribuzin [4-amino-6-tert-butyl-3-methylsulfanyl-1,2,4-triazin-5-one] belongs to systemic triazine herbicides and is a photosystem II inhibitor; it is used in potatoes, tomatoes, soybean, and carrot [27]. Halosulfuron methyl (methyl 3-chloro-5-[(4,6-dimethoxypyrimidin-2-yl)carbamoylsulfamoyl]-1-methylpyrazole-4-carboxylate) belongs to the sulfonylureas and is an inhibitor of the enzyme acetolactate synthase (ALS) [28]. The herbicide is selective and systemic [29]. Flumioxazin (2-(7-fluoro-3-oxo-4-prop-2-ynyl-1,4-benzoxazin-6-yl)-4,5,6,7-tetrahydroisoindole-1,3-dione) is a N-phenylphalimide [30] and is an inhibitor of the enzyme protoporphyrinogen oxidase (PPO or Protox) [31]. The stock solutions and dilution series of metribuzin, halosulfuron were prepared in distilled water (1100 and 1630 mg L^−1^) and for flumioxazin (1.7 mg L^−1^) in acetone [24]. Acetone was used for flumioxazin due to its low solubility in water (if soluble to less than a concentration of 1000 mg L^−1^). The dilution series was made just before the application. A water control was included metribuzin and halosulfuron herbicides. A positive control (using the same amount of distilled water as added in each test substance assay) and a negative control (using the same amount of acetone for solvent control) were also tested for flumioxazin [24].

### 2.3. Toxicity Test Methods

#### 2.3.1. Filter Paper Test

An 8 cm glass Petri dish was lined with filter paper (Whatman filter paper no. 1) cut into 8 × 3 cm diameter without overlapping [24]. Metribuzin and halosulfuron were dissolved in distilled water, while flumioxazin was dissolved in acetone. Two-milliliter solutions were pipetted into the Petri dish The concentrations of metribuzin, halosulfuron, and flumioxazin applied singly are shown in Table 1.

Control Petri dishes were also run in parallel with the herbicides. Distilled water was used as the control for metribuzin and halosulfuron and distilled water and acetone for flumioxazin. The acetone took around one hour to evaporate. After the acetone was evaporated in an airing chamber, well-developed clitellate adult worms were randomly selected, washed, and dried. Then, they were exposed (one earthworm per Petri dish) to 2 mL of different concentrations of herbicides for 24 and 48 h [24]. The earthworms were kept on wet filter paper for 24 h at 20 ± 1 °C in the dark to have the gut contents purged before the dose–response test and washed and dried before being weighed and introduced in the test [32]. The filter paper test had three replications. The ten adult earthworms and Petri dishes were used per replication. Every Petri dish contained one adult earthworm [24]. Petri dishes were kept in a 16:8 light:dark regime under 400–800 lux of constant light and 20 ± 1 °C at 80–85% relative humidity and covered with a plastic lid with holes to allow for aeration and prevent the earthworms from escaping the Petri dishes. The experiments were performed for a 48 h treatment period, and the number of dead earthworms in each treatment was recorded on each Petri dish [24]. A preliminary range-finding test to find an optimal dose range that caused 0–100% mortality was performed for the single herbicides before the mixture experiments (data not shown) [24].

#### 2.3.2. Soil Toxicity Test

An artificial soil consisted of 10% ground sphagnum peat (<0.5 mm), 20% kaolin clay (>45% kaolinite), 70% quartz sand (<0.2 mm), and a small amount of calcium carbonate to adjust the soil pH at 6.0 ± 0.5 [24]. The water content was adjusted to 50%. The concentrations of metribuzin, halosulfuron, and flumioxazin applied singly are shown in Table 1. The stock solution of metribuzin and halosulfuron was prepared in water while flumioxazin was dissolved in acetone. This stock solution was used to spike soil with the highest test concentration and further diluted for spiking soil with the lower concentrations tested. The desired amount of herbicides was dissolved in 10 mL distilled water or acetone and mixed into a small quantity of fine quartz sand for each concentration. The sand was mixed for at least one hour to evaporate the acetone and then mixed thoroughly with the pre-moistened artificial soil in a household mixer. All soils were thoroughly mixed to achieve a homogenous distribution of the herbicides. The addition of distilled water adjusted the final moisture contents of artificial soil. About 0.650 kg of artificial soil (equivalent to 0.5 kg dry artificial soil) was added into a 1 L glass jar for replicate. The soil test had three replications. Three glass jars, each containing ten adult earthworms, were used for each concentration. Positive and negative controls were prepared with 10 mL distilled water or acetone and no herbicide to ensure that the positive and negative controls were not significantly different from each other. The earthworms were removed from the culture, rinsed with water, and placed on damp filter paper in the dark at 20 ± 1 °C, and the content of their guts was emptied 24 h before herbicide exposure [32]. The earthworms were fed 5 g cattle manure. Test jars were weighed at the start, so water loss could be monitored weekly and replenished with deionized water if needed. All earthworms were weighted before the test. The jars were covered with perforated plastic film to allow air exchange and kept in a 16:8 light:dark regime under 400–800 lux of constant light at room temperature (20 ± 1 °C) with 80–85% relative humidity. The survival was recorded at 1, 7, and 14 days after the treatment.

The earthworms were preconditioned for 24 h under the same conditions described above in the untreated soil before the dose–response test. The ten adult earthworms were purged for 24 h and were washed and dried before being weighed and introduced in the test. Two preliminary range-finding tests determined the concentration range of the herbicides to find an optimal dose range to cause 0–100% mortality (data not shown) [24].

#### 2.3.3. Mixture Toxicity

Based on the measured LC_50_ values of the individual herbicides (A and B) on filter paper and artificial soil tests (Equation (1)), the relative potency (r) (Equation (2)) was determined for the herbicides applied alone on filter paper and artificial soil tests. LC_50_s concentration (µg ai. cm^−2^ or mg ai. kg^−1^) reduced the live earthworm number by 50%. The LC_50_s were derived from the dose–response regression model (Equation (1)) based on Equation (2); the fixed mixture ratios were determined to ensure evenly distributed LC_50_ values for mixtures along the CA isobole [33]. (Data not shown). The mixture ratios were (100:0), (10:90), (25:75), (50:50) and (0:100)% for the metribuzin:halosulfuron mixtures and for metribuzin:flumioxazin, they were (100:0), (4:96), (12:88), (29:71) and (0:100)% on the filter paper test. The dose–response mixture experiments were independently repeated twice. As with the filter paper test, the mixtures ratios for the soil test were based upon the relative potencies of the individual herbicides. For metribuzin:halosulfuron, the mixtures were (100:0), (72:28), (46:54), (22:88) and (0:100)%, and for metribuzin:flumioxazin, they were (100:0), (25:75), (10:90), (4:96) and (0:100)% [34]. A positive water control was tested for metribuzin:halosulfuron and there were two water and acetone controls for metribuzin:flumioxazin for the filter paper and artificial soil tests. In neither case were there significant differences between the positive control water only and negative control water plus acetone treatments.

### 2.4. Statistical Analysis

The dose–response data were analyzed using the R program (Version 3.6.1). The survival of earthworms in response to herbicides is classical in toxicology. The binomial response, dead or alive, was assessed at various times during the experiments is fundamental for classifying toxic compounds. The log-logistic regression was used to determine the acute toxicity of metribuzin, halosulfuron, and flumioxazin on filter paper and artificial soil tests (Equation (1)). The add-on R package drc (Version 3.6.1) was used to fit the log-logistic curves.

The non-linear regression analysis of the log-logistic model [35] is seen below.

(1) $$y=\frac{1}{1+{\left(\frac{x}{{\mathrm{LC}}_{50}}\right)}^{b}}$$

where y is the binomial response, alive divided by the total number of earthworms in a Petri dish or artificial soil, x denoted herbicide concentration (µg ai. cm^−2^ or mg ai. kg^−1^) of individual herbicides. LC_50_ is the concentration (µg ai. cm^−2^ or mg ai. kg^−1^) that kills 50% of the total earthworms, and b is the slope of the curve around LC_50_. The dose–response fitted reasonably well to the data by a lack-of-fit test. The relative potency (r) (Equation (2)) was determined for the herbicides applied alone on filter paper and artificial soil tests. Equation (2) expresses the biological exchange rate between the herbicides, metribuzin, halosulfuron, and flumioxazin within the filter paper and the soil toxicity test.

(2) $$r={\mathrm{LC}}_{50A}/{\mathrm{LC}}_{50B}$$

The LC_50_s were derived from the dose–response regression model (Equation (1)) based on Equation (2); the fixed mixture ratios were determined to ensure evenly distributed LC_50_ values for mixtures along the CA isobole [33,34] (data not shown).

The non-linear regression analysis of the log-logistic model was used to determine the acute toxicity of metribuzin:halosulfuron and metribuzin:flumioxazin mixture ratios (Equation (1)). The dose–response fitted reasonably well to the data by the lack-of-fit test. The LC_50_ parameters for each curve of fixed-ratio mixtures illustrated the deviation from the straight-line isobole of the CA reference model [35]. The reference model was the Concentration Addition (CA) is also called the Additive Dose Model (ADM) [20,22]. Deviation of the mixture isoboles from the straight-line CA isoboles was used to classify the toxicology of those mixtures on earthworm populations in two test systems. The mixture experiments were repeated twice to substantiate if the mixture deviation from the CA was consistent. The isobole method can be used to calculate the joint action of herbicides. The mixtures of the metribuzin:halosulfuron and metribuzin:flumioxazin reference model CA at the equivalent LC_50_ doses can be expressed by [36]:

(3) $$Z_{1}{=\mathrm{rZ}}_{2}{=z}_{m\;}{=z}_{1\;}{+\mathrm{rz}}_{2\;}\;$$

where Z_1_ and Z_2_ are the LC_50_ of herbicide 1, and 2 applied singly, and z_1_ and z_2_ are the LC_50_ of herbicide 1 and 2 in a mixture adjusted by the relative potency, r (Equation (2)) herbicide 1 and 2 is applied alone in Equation (2). In an LC_50_ isobologram, the X- and Y-axes are the dose axes of each herbicide in a mixture. Metribuzin is the dose of the X-axis, and halosulfuron or flumioxazin is the dose on the Y-axis. The solid lines for each LC_50_ connecting the herbicides applied singly are the theoretical CA isobole. If the herbicides in a mixture do not interact, the mixtures follow the straight-line isobole and thus comply with the CA reference model (Equation (3)). When herbicides are less effective than expected, they show antagonistic action: this means that one must use higher doses of each herbicide in a mixture to produce the same effect as that of the herbicides applied alone. When herbicides are more effective than expected, they show synergistic action: this means that one must use lower doses of each herbicide in a mixture to produce the same effect as that of the herbicides applied alone.

Student *t*-test was applied to compare the results from the controls with and without acetone for each experiment. No significant differences were found for the tests (*p* value > 0.05).

## 3. Results

### 3.1. Filter Paper Test

Table 2 shows the LC_50_ of the herbicides applied singly taken at various times after herbicide applications and the test for lack of fit of the dose–response curves. Only the metribuzin reading at 24 h had a significant lack of fit. The LC_50_ declined between 24 and 48 h for metribuzin and flumioxazin. The toxicity of herbicides was ranked as metribuzin > halosulfuron > flumioxazin, and the ranking did not change between the time of measurement (Table 2). The LC_50_ of halosulfuron was the same 52.95 µg ai. cm^−2^ at 24 and 48 h. The test for lack of fit [33] was significant for metribuzin at 24 h but alarmingly small in any other instances. The lack of fit test is relatively weak and was supported by compared residual plots (data not shown) [33]. The LC_50_ estimates were not significantly different for water control and acetone control for flumioxazin at both timepoints (Student *t*-test; *p* = 0.374 on 24 and *p* = 0.158 at 48 h).

**Figure 1:**
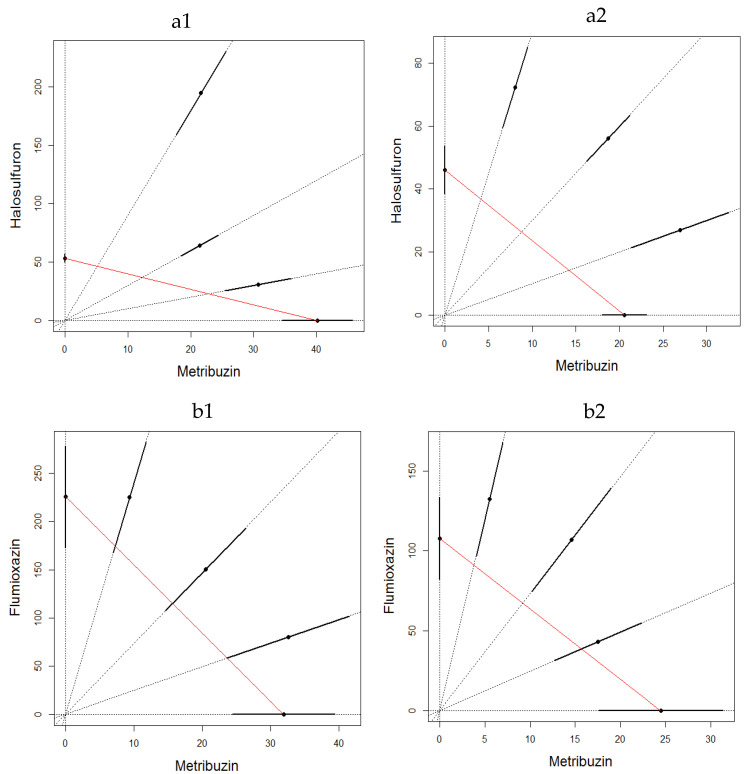
LC_50_ isobologram showing the toxicological interactions of metribuzin:halosulfuron (**a**) and metribuzin:flumioxain (**b**) on *Eisenia fetida* in first experiment of filter paper test at 24 h (1) and 48 h (2). The straight line of isobologram indicates additivity. The lines around the mixture points are 95% confidence intervals.

**Figure 2:**
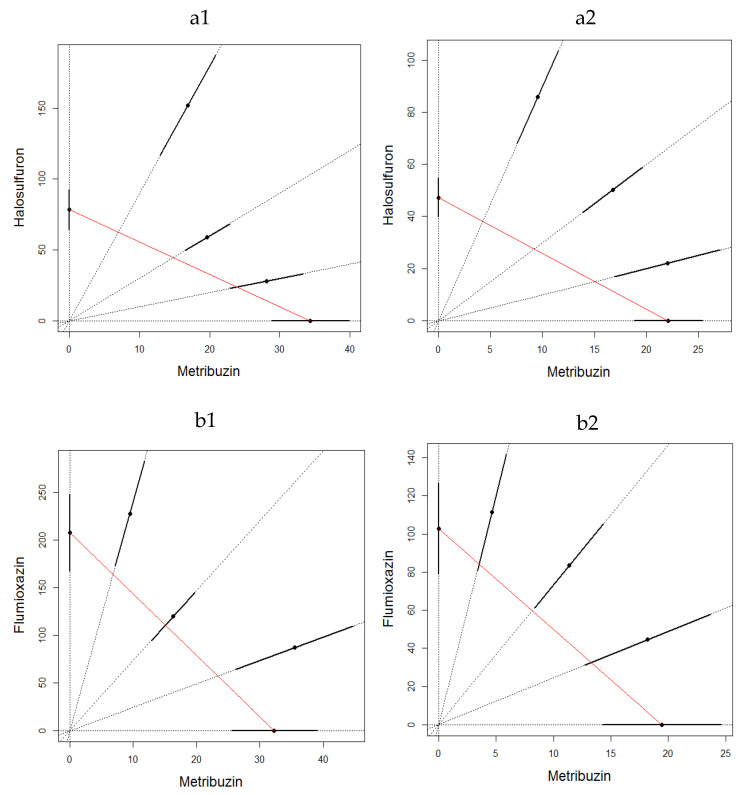
LC_50_ isobologram showing the toxicological interactions of metribuzin:halosulfuron (**a**) and metribuzin:flumioxain (**b**) on *Eisenia fetida* in the second experiment on filter paper test at 24 h (1) and 48 h (2). The straight line of isobologram indicates additivity. The lines around the mixture points are 95% confidence intervals.

The binary mixture experiments demonstrated for both experiments an apparent antagonistic effect at both sampling times and for both binary mixtures (Figure 1 and Figure 2). The LC_50_ estimates were not significantly different for water control and acetone control for metribuzin:flumioxazin in both experiments (Student *t*-test; *p* = 0.116 on 24 and 48 h in the first experiment and *p* = 0.374 on 24 and *p* = 0.205 on 48 h in the second experiment). It is also important to emphasize that the mixture ratios were almost evenly distributed along the CA isoboles.

### 3.2. Soil Toxicity Test

Earthworms exposed to metribuzin, halosulfuron, and flumioxazin also showed changes in LC_50_ in sampling time. The results demonstrated that an increase in exposure time decreased the LC_50_, particularly for flumioxazin **(**Table 2**)**. The test for lack of fit was in no instance significant, meaning that the dose–response curves fitted reasonably to the data. The LC_50_ estimates were not significantly different for water control and acetone control for flumioxazin (Student *t*-test; *p* = 0.374 on 1, 7, and 14 days). The toxicity of herbicides was ranked as metribuzin > halosulfuron > flumioxazin (Table 2), and the ranking did not change among the time of measurement.

**Figure 3:**
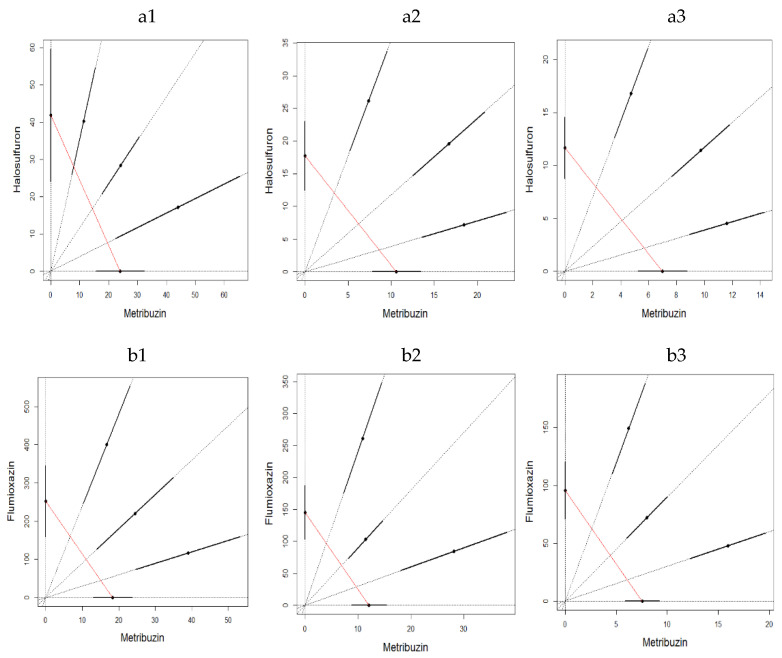
LC_50_ isobologram showing the toxicological interactions of metribuzin:halosulfuron (**a**) and metribuzin:flumioxazin (**b**) on *Eisenia fetida* in the first experiment of artificial soil test on 1 day (1), 7 days (2) and 14 days (3). The straight line of isobologram indicates additivity. The lines around the mixture points are 95% confidence intervals.

**Figure 4:**
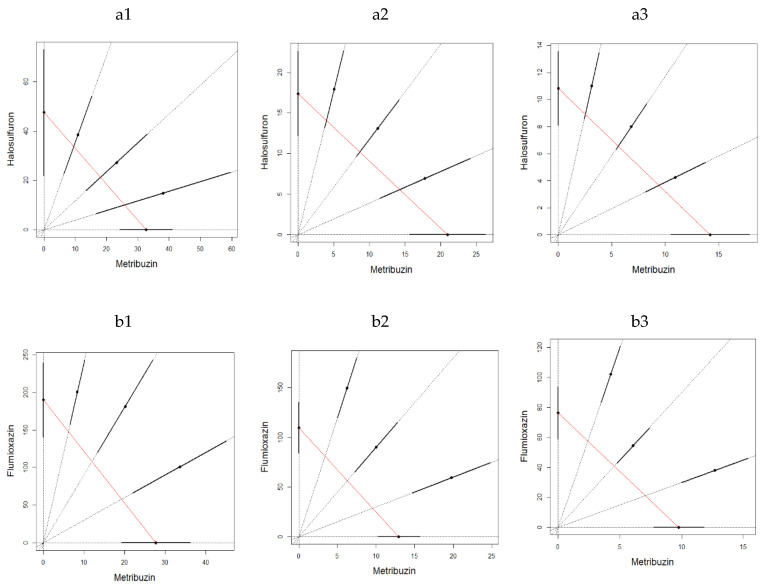
LC_50_ isobologram showing the toxicological interactions of metribuzin:halosulfuron (**a**) and metribuzin:flumioxazin (**b**) on of *Eisenia fetida* in the second experiment of artificial soil test at 1(1) day, 7 (2) days and 14 (3) days. The straight line of isobologram indicates additivity. The lines around the mixture points are 95% confidence intervals.

The binary mixture experiments demonstrated, for both experiments, a clear antagonistic effect at all three sampling times and for both binary mixtures (Figure 3 and Figure 4). The LC_50_ estimates were not significantly different for water control and acetone control for metribuzin:flumioxazin at different mixture ratios in both experiments (Student *t*-test; *p* = 0.374, 0.116 and 0.116 on 1, 7, and 14 days in the first experiment and *p* = 0.374, 0.374, 0.158 on 1, 7 and 14 days in the second experiment). It is also important to emphasize that the mixtures ratios were almost evenly distributed along the CA isoboles.

## 4. Discussion

Mortality of *Eisenia fetida* is typically used in studying chemical toxicity compounds on earthworms [37,38]. The contact filter paper test is a fast, simple, and inexpensive test to screen for toxicity [10]. It also applies to the soil test, but it is obvious that the potency of the herbicides and herbicide mixtures were notorious different between the two test media. However, the LC_50_ gave the same order of toxicity metribuzin > halosulfuron > flumioxazin. The herbicides are designed for weed control; therefore, the sites and modes of action are well known in plants but not in animals. When it comes to the site and mode of action in animals, the cause-and-effect relationships between mortality and specific action sites become more uncertain and need to be further investigated.

The increase in exposure time decreased the LC_50_ in both test systems. For metribuzin and halosulfuron, 100% mortality at high doses was observed 3–4 h after exposure on filter paper. The LC_50_ of the PS II inhibitor, metribuzin, had the highest toxicity in both test systems. This means that the compound may have some unknown effect on the earthworm. On the other hand, flumioxazin, with its particular site of action on heme and chlorophyll metabolism, is known to affect the soil biome [39].

Chemicals with LC_50_ values of 10–100 µg cm^−2^ on filter paper were classified as ‘‘very toxic’’ [40]. According to this standard, metribuzin and halosulfuron toxicity to earthworms can be classified as high. However, the halosulfuron, an ALS inhibitor, is used at small field rates, so perhaps the effect of this herbicide in the field is not so severe as is metribuzin.

Herbicides affected the earthworms adversely through skin contact. Earthworm mortality by the presence of metribuzin was caused by increased mucous secretion to a high concentration (laboratory observations). The earthworms exposed to metribuzin exhibited surface wounds and extrusion of coelomic fluid. It caused bloody lesions on the posterior part of the body and ultimately death. Fragmentation of the body was also observed (laboratory observations). The single and binary mixtures applied to the soil test, but it is obvious that the potency of the herbicides and herbicide mixtures different between the two test media. The ranking of the herbicides was the same in both test systems with rather low metribuzin and halosulfuron LC_50_ compared to flumioxazin, particularly in the soil test. However, we have to bear in mind that the field rates of metribuzin, halosulfuron and flumioxazin are 700–1000, 250 and 100 g ha^−1^. The (50:50) and (25:75)% mixture ratios of metribuzin:halosulfuron and metribuzin:flumioxain provided higher toxicity than the other mixture ratios (100:0) and (0:100)% on earthworm biomass, respectively. Isobologram demonstrated that metribuzin:halosulfuron and metribuzin:flumioxazin followed an antagonistic effect, meaning that the mixtures retracted the action of the herbicide in the earthworms relative to a Concentration Addition (CA) reference model on earthworm biomass [2].

Mortality is related to an earthworm strategy for decreasing food consumption to avoid toxins. This strategy is used for heavy metals [41] and pesticides [10]. Soil ingestion and dermal absorption are the most crucial uptake paths of soil contaminants by earthworms. Earthworms can absorb and accumulate pollutants in their body tissues through the skin and digestive system [42]. On the other hand, they may induce cytochrome P_450_. Cytochrome P_450_ monooxygenases play a main role in metabolism herbicides [43,44].

It should also be noted that increasing mortality might correlate with the high persistence of metribuzin and halosufuron in soil or the slow degradation in the earthworms and, subsequently, less metabolite elimination. Researchers have documented that flumioxazin has a soil half-life between 11.9 and 17.5 days [29]. A short half-life influences the toxicity of a compound, particularly in the artificial soil experiment performed here.

While the herbicides were well mixed in the soil test, in the field, most of the herbicide residues were found in the upper 5 cm of the profile [45]. The results also indicated the low toxicity of flumioxazin in filter paper and artificial soil tests and could be attributed to the rapid elimination in the animals. Travlos et al. [5] investigated the survival of *E. fetida* in soil toxicity bioassays of benfluralin, metribuzin and propyzamide. They reported that the highest mortality was found after the treatment with double the recommended field rate of metribuzin (1500 g ai. ha^−1^) at three weeks after treatment. However, this is only a single field rate that cannot be referred to the test systems used here.

The classic model for predicting mixture toxicity, such as Concentration Addition (CA), is based on simple assumptions on the mode of toxic action [20,22,46]. However, synergism or antagonism can occur irrespective of the primary site and mode of action [47]. Detracted action, also denoted as antagonistic action, was rather consistent in the experiments. It means that to obtain the same effect level (e.g., LC_50_), one needs a higher concentration of the mixture than that of the individual compounds applied singly. The results were indeed confirmed as we did two independent experiments. The antagonistic response might result in the reduced toxicity on earthworms [48]. There is no evidence of metribuzin:halosulfuron and metribuzin:flumioxazin interaction in the literature.

Our results of the filter paper test are in line with the artificial soil test of individual and combined herbicides toxicity and confirmed the work by [49]. It is well known that the laboratory test results cannot be extrapolated to the field [50,51]. However, in some rare cases, the field and laboratory outcomes are comparable [52,53,54]. The complementarity between field investigations and laboratory tests is a perpetual discussion in the literature. When introducing various mixtures, one needs both methods, as noted by [55].

## 5. Conclusions

The results in our experiments demonstrated that the ranking of the toxicity of the herbicides was the same whether we used the filter paper test or soil test, and the lethality increased in the course of time of exposure. The herbicide mixtures all consistently acted antagonistically whether the mortality was assessed 24 or 48 h or up to fourteen days after the initiation of the experiments on filter paper and in soil test, respectively.

## Figures and Tables

**Table 1:** Concentration used in filter paper and artificial soil tests.

**Herbicide**	**Concentration for Filter Paper Test (µg ai. cm^−2^)**
Metribuzin	0, 0.156, 0.312, 0.625, 1.25, 2.5, 3.5, 5, 7, 10, 14, 28, 56, 112, 224
Halosulfuron	0, 0.0156, 0.0312, 0.0625, 0.125, 0.250, 0.500, 1, 2, 4, 8, 16, 32, 64, 128, 256
Flumioxazin	0, 10, 20, 40, 80, 160, 320, 640
	**Concentration for artificial soil test (mg ai. kg^−1^)**
Metribuzin	0, 0.359, 0.718, 1.436, 2.872, 5.744, 11.488, 22.976
Halosulfuron	0, 0.2051, 0.410, 0.820, 1.640, 3.281, 6.563, 13.126
Flumioxazin	0, 4.1026, 8.205, 16.41, 32.82, 65.64, 131, 262

**Table 2:** Estimated sigmoidal parameters for metribuzin, halosulfuron and flumioxazin in filter paper and artificial soil tests. Standard errors in parentheses.

Filter paper												
Herbicide	LC_50_ (µg ai. cm^−2^)						Lack of fit					
	24 h			48 h			24 h			48 h		
Metribuzin	34.00 (2.23)			17.17 (1.17)			0.003			0.97		
Halosulfuron	52.95 (9.58)			52.95 (9.58)			1			1		
Flumioxazin	241.64 (31.62)			127.42 (16.61)			0.08			0.06		
Artificial soil												
Herbicide	LC_50_ (mg ai. kg^−1^)						Lack of fit					
	1 day		7 days		14 days		1 day		7 days		14 days	
Metribuzin	25.75 (12.44)		11.72 (4.36)		8.48 (3.36)		0.95		0.95		0.08	
Halosulfuron	27.63 (13.38)		14.26 (4.26)		10.08 (2.76)		0.97		0.98		0.94	
Flumioxazin	315.09 (115.05)		157.67 (40.49)		74.84 (13.79)		0.95		0.94		0.42	

## Data Availability

Data are available by contacting ESK (samadielham@uma.ac.ir).
